# Can Digenic, Tri-Allelic Inheritance of Variants in *STAR* and *CYP11A1* Give Rise to Primary Adrenal Insufficiency? A Case Report

**DOI:** 10.3389/fendo.2022.860055

**Published:** 2022-03-28

**Authors:** Naseer Ali, Avinaash Vickram Maharaj, Federica Buonocore, John C. Achermann, Louise A. Metherell

**Affiliations:** ^1^ Department of Endocrinology and Metabolism, Meitra Hospital, Calicut, India; ^2^ Centre for Endocrinology, William Harvey Research Institute, Queen Mary, University of London, London, United Kingdom; ^3^ Genetics & Genomic Medicine, UCL Great Ormond Street Institute of Child Health, University College London, London, United Kingdom

**Keywords:** primary adrenal insufficiency, isolated glucocorticoid deficiency, STAR, CYP11A1 (P450scc), puberty

## Abstract

An eight-year old South Asian boy presenting with progressive hyperpigmentation was found to have primary adrenal insufficiency (PAI) in the form of isolated glucocorticoid deficiency. Follow up of this boy for nine years, until the age of 17 years showed normal pubertal onset and progression. Molecular evaluation, by targeted next generation sequencing of candidate genes linked to PAI revealed changes in two genes that are intricately linked in the early stages of steroid biosynthesis: compound heterozygous variants in *STAR*, c.465+1G>A and p.(E99K), plus a heterozygous rs6161 change in *CYP11A1*. No variants in other known causal genes were detected. The proband’s mother was heterozygous for the c.465+1G>A *STAR* and rs6161 *CYP11A1* variants, while the father was homozygous for the p.(E99K) alteration in STAR but wild-type for *CYP11A1*. Both parents had normal adrenal cortical function as revealed by short Synacthen tests. The *STAR* variant c.465+1G>A will lead to abnormal splicing of exon 4 in mRNA and the addition of the p.(E99K) variant, predicted damaging by SIFT and CADD, may be sufficient to cause PAI but this is by no means certain given that the unaffected father is homozygous for the latter change. The rs6161 *CYP11A1* variant [c.940G>A, p.(E314K)] has recently been demonstrated to cause PAI in conjunction with a severe rare disruptive change on the other allele, however sequencing of the coding region of *CYP11A1* revealed no further changes in this subject. We wondered whether the phenotype of isolated glucocorticoid deficiency had arisen in this child due to tri-allelic inheritance of a heterozygous *CYP11A1* change along with the two *STAR* variants each of which contribute a partial loss-of-function burden that, when combined, is sufficient to cause PAI or if the loss-of-function c.465+1G>A combined with the presumed partial loss-of-function p.(E99K) in *STAR* could be causative.

## Introduction

Congenital primary adrenal insufficiency (PAI) in children is usually inherited as one of numerous monogenic disorders, with a molecular diagnosis being made in more than 90% of individuals (1–3). Principal causes include congenital adrenal hyperplasia (CAH), X-linked congenital adrenal hypoplasia (AHC), familial glucocorticoid deficiency (FGD) caused by adrenal cortical unresponsiveness to ACTH, adrenoleukodystrophy (ALD) and triple A syndrome (Allgrove syndrome) (1, 2). More recently, next generation sequencing (NGS) of patients with PAI has identified mutations in genes linked to altered redox potential, impaired oxidoreductase activity and sphingolipid metabolism, which include minichromosome maintenance 4 (*MCM4*), nicotinamide nucleotide transhydrogenase (*NNT*), thioredoxin reductase 2 (*TXNRD2*) and sphingosine 1-phosphate lyase (*SGPL1*) (4–7). Reaching a specific diagnosis can have implications for management and counselling. The genetic causes of PAI and their spectrum of disease have been reviewed recently (2, 3, 8).

FGD usually presents with isolated glucocorticoid deficiency with minimal or no mineralocorticoid deficiency. Inactivating mutations of the type 2 melanocortin receptor gene (*MC2R*) were the first genetic cause to be associated with FGD (FGD 1) (9, 10). Later, mutations in melanocortin 2 receptor accessory protein (*MRAP*) were also found to be causative (FGD2) (10–12). Together pathogenic variants in these genes only account for less than half of all cases of FGD.

Although mutations in steroidogenic acute regulator protein (*STAR*) have classically been associated with congenital lipoid adrenal hyperplasia, characterized by severe deficiency of both glucocorticoids and mineralocorticoids and no androgenization of 46,XY fetuses (13), partial loss-of-function variants in *STAR* are increasingly reported in patients diagnosed with FGD who presented with later onset, milder disease, and apparently normal gonadal function (14–16). *CYP11A1* encodes the P450 side chain cleavage enzyme (P450scc) crucial in early steroidogenesis and, similar to *STAR*, classical *CYP11A1* mutations (resulting in disruption of the enzyme, P450scc) also cause severe deficiency of both glucocorticoids and mineralocorticoids with no androgenization of 46,XY fetuses. Recently, certain mutations in *CYP11A1* have also been found in patients with milder phenotypes indistinguishable from FGD (17, 18); indeed, several studies have reported patients with the combination of a rs6161 (c.940G>A) variant that affects splicing, together with a severe loss-of-function change on the other allele as a cause of PAI presenting in childhood, but with normal or only mildly disrupted gonadal function (18). Taken together with the emerging experience from non-classic congenital lipoid adrenal hyperplasia due to *STAR* defects, these reports highlight a relation between enzyme function and phenotype and suggest that human adrenal function is more sensitive to partial disruption of *STAR* and *CYP11A1* than gonadal (testis) function.

Here we report long-term follow up of a boy with PAI presenting as isolated glucocorticoid deficiency possibly due to tri-allelic inheritance of variants in *STAR* and *CYP11A1*.

## Case Report

A boy born to non-consanguineous South Asian parents presented at the age of eight years with a history of progressive hyperpigmentation since early childhood. There was no prior history of electrolyte disturbances or evidence of adrenal crisis and family history was unremarkable for adrenal insufficiency. Additionally, screening for tuberculosis was negative. On examination, height was 124.5 cm (-0.6 SDS), target height 166 cm (-1.5 SDS) and weight 21 kg (-1.5 SDS). He had male external genitalia with no hypospadias, testicular volume of 2 ml bilaterally and absent pubic or axillary hair (Tanner stage 1). Blood pressure was 98 mmHg systolic and 66 mmHg diastolic. Hyperpigmentation of the skin was evident. There were no features suggestive of neurodegeneration, alacrima or achalasia, or other systemic features of note.

Serum biochemistry obtained at 8 years of age is presented in **Table 1**. His 8AM serum cortisol was within the lower normal range but was inappropriately low given the massively elevated ACTH. CT scan showed bilateral small adrenal glands. The patient was treated with oral hydrocortisone (7mcg/m^2^/day) which led to significant improvement in general well-being and partial improvement in hyperpigmentation. Neither parent manifested signs or symptoms of adrenal insufficiency and both had a normal cortisol response to short standard Synacthen tests (father, 49 years of age, post Synacthen cortisol 28.4 mcg/dL; mother, 43 years of age, post Synacthen cortisol 25.9 mcg/dL; with a post Synacthen cortisol > 20 mcg/dl considered as an adequate response).

**Table 1:** Biochemical investigations at initial presentation at 8 years of age.

	Value	Reference range
Serum Sodium	138 mEq/L	135-145 mEq/L
Serum Potassium	4.4 mEq/L	3.5-5 mEq/L
Serum Cortisol (8AM)	6 mcg/dL	6-23 mcg/dL
Plasma ACTH (Adrenocorticotropic hormone)	>2000 pg/mL	5-50 pg/mL
Serum DHEAS (Dehydroepiandrosterone Sulphate)	5.7 mcg/dL	23 - 209 mcg/dL
Plasma Renin activity	9.63 ng/mL/Hour	1.9- 5.2 ng/mL/Hour
Plasma Aldosterone concentration	121.8 pg/mL	12- 340 pg/mL

After 5 years of follow up (at age 13), the proband’s testicular volume was 6 ml bilaterally, pubic hair stage Tanner P1, and his height was 142 cm (-1.85 SDS), indicating age appropriate physiological onset of puberty. On examination at the age of 16 years, he had progressed through puberty; his height was 167.5 cm (-0.80 SDS), weight 52.6 kg (-0.92 SDS), blood pressure 106/60 mmHg. His testicular volume was 20 ml on both sides, pubic hair tanner stage P3. His morning serum testosterone level was 6.98 ng/mL (normal adult range, 2.3 - 9.5 ng/mL), and serum luteinizing hormone (LH) (2.73 IU/mL), and follicle-stimulating hormone (4.71 IU/mL) were normal. Further follow up at the age of 17 years confirmed a testicular volume of 20 ml on both sides, and showed progressive hair growth with pubic hair Tanner stage 5, and axillary hair present. His height was 170.5cm (-0.66 SDS). He had adequate height gain during puberty, so that his height SDS improved by the age of 17 years. He has a younger brother (10 years of age), who is asymptomatic and not hyperpigmented, and did not consent for hormonal or molecular testing.

### Molecular Analysis

**Figure 1:**
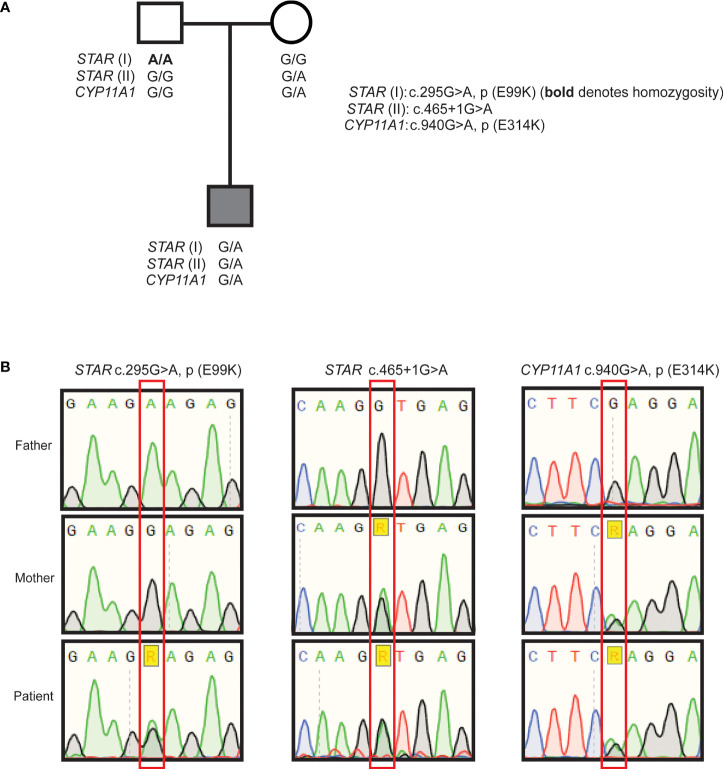
Pedigree of the patient and Sanger sequencing results. Family pedigree indicating inheritance of the variants **(A)**, partial chromatograms from Sanger sequencing of *STAR* variants p.(E99K), c.465+1G>A, and *CYP11A1* rs6161; c.940G>A **(B)**.

The proband was further evaluated for putative pathogenic variants in causal FGD genes and no variants were found in *MC2R* and *MRAP*. Further sequencing was performed on a HaloPlex targeted NGS panel (19) enabling concomitant detection of any variants in genes associated with PAI. Analysis showed that the proband had two heterozygous variants in STAR: chromosome 8:38003806C>T; c.465+1G>A and 8:38005729C>T; c.295G>A (rs903915397); p.(E99K) (GRCh37) (**Figures 1A, B**). Neither change has been previously reported in association with PAI and both are extremely rare. The c.465+1G>A change is not present in the gnomAD variant database, and the p.(E99K) change is very rare with only one heterozygous individual identified in gnomAD (minor allele frequency of 0.00003267 in South Asians). The c.465+1G>A variant was inherited from the boy’s mother, and is a canonical splice site mutation at the junction of exon 4 and intron 4. This variant alters the canonical donor splice site of intron 4 and is likely to lead to the skipping of exon 4 with the predicted consequence for the protein being an in-frame deletion of 56 amino acids (p.103_155del). The other *STAR* variant, c.295G>A; p.(E99K) is predicted to be damaging by SIFT (score 0.01) and CADD (score 27.5). His father who is homozygous for this variant, did not manifest an adrenal phenotype suggesting at least some residual function. The patient also inherited a heterozygous change, rs6161, in *CYP11A1* [c.940G>A, p.(E314K)] from his mother (**Figures 1A, B**), which was recently shown to result in partially defective splicing thereby producing a non-functional protein (18, 20). A second variant in *CYP11A1* was not identified. No other variants in genes on the HaloPlex array and known to cause adrenal insufficiency were uncovered by this analysis. This combination of findings raises the possibility that a non-classical congenital (lipoid) adrenal hyperplasia is the cause of isolated glucocorticoid deficiency in this patient due to combined digenic, tri-allelic inheritance of two *STAR* mutations and the rs6161 *CYP11A1* variant.

## Discussion

This case describes the follow up of a boy with PAI, presenting as isolated glucocorticoid deficiency with small adrenals, possibly due to tri-allelic inheritance of two *STAR* mutations and the rs6161 *CYP11A1* variant. Although classically individuals with *STAR* mutations reportedly have large lipid laden adrenal glands in early life, there are reports of individuals with small adrenals, which may sometimes be due to the longer term effects of adrenal damage (21–23). In non-classic congenital lipoid adrenal hyperplasia, due to partial loss-of-function of *STAR*, the picture is mixed with both normal sized and hypoplastic adrenals described (14, 16, 24). For classic, loss-of-function mutations in *CYP11A1* large adrenal glands have not been described, but more often small or absent adrenals are noted (25) and - similar to *STAR* - partial loss-of-function variants of P450scc are associated with normal or small but never enlarged adrenal glands (18, 26, 27). Hence, whilst enlarged adrenals could have indicated a complete loss-of-function of *STAR*, normal or even small sized adrenals cannot rule out *STAR* or *CYP11A1* defects and give us no clue here to the underlying cause.

*STAR* mutations usually give rise to congenital lipoid adrenal hyperplasia but partial loss-of-function variants can present with an FGD-like phenotype of isolated glucocorticoid deficiency. Indeed, Baker et al. (16) described three FGD phenotype patients with STAR p.(V187M) and p.(R188C) mutations. Structural and functional analysis of these mutants revealed that they retained greater than 20% cholesterol binding activity. This residual activity may be the reason for the mild phenotype in these patients. Other studies also demonstrated that certain mutations in STAR [p.(R192C) and p.(R188C)] can present with a phenotype similar to that of FGD (13). In our patient, the *STAR* variant c.465+1G>A is predicted to lead to the skipping of exon 4 of the transcript with consequent in-frame deletion of 56 amino acids (p.103_155del) in the protein. This variant is likely to be loss-of-function. The second STAR variant p.(E99K) is predicted to be damaging, however the father of the patient was homozygous for this variant and did not manifest disease, suggesting only partial loss-of-function. Classical *CYP11A1* mutations also cause severe deficiency of both glucocorticoids and mineralocorticoids and no androgenization of 46,XY fetuses. Partial loss-of-function variants in *CYP11A1* can also give rise to a mild phenotype of a predominant FGD phenotype, with variable mineralocorticoid deficiency and often preserved testicular function (17). Indeed, the *CYP11A1* rs6161 variant found in our patient, which was initially considered to be benign and has a minor allele frequency in gnomAD of 0.002561 (0.001592 in South Asians), has recently been found to cause isolated glucocorticoid deficiency when combined with a severe loss-of-function *CYP11A1* defect, especially in patients from a European lineage (18, 20). This variant has been shown to have partial loss-of-function through mis-splicing and a reduced half-life of the protein P450scc (18, 28). We were unable to identify a second *CYP11A1* variant in the patient, suggesting a P450scc defect alone is not causal. We hypothesize that the combination of a null allele (p.103_155del) with a partial loss-of-function allele [p.(E99K)] in STAR along with reduced function of P450scc due to the *CYP11A1* variant results in the phenotype; in effect, that sub-optimal function of the STAR protein, with biallelic inheritance of the two variants, alongside haploinsufficiency of p450scc side chain cleavage (*CYP11A1*) can be enough to reduce steroidogenic output such that the patient has late onset glucocorticoid deficiency but preserved mineralocorticoid and gonadal steroid production. This would be akin to the situation seen with non-classical congenital lipoid adrenal hyperplasia due to *STAR* variants p.(R188C) & p.(R192C) (14, 16) and non-classic P450scc deficiency due to mutations such as p.(R451W), p.(L222P), c.940G>A (rs6161) & p.(A269V) (26, 27, 29).

This young man appeared to progress through puberty. Individuals with partial defects in P450scc can sometimes later develop abnormal germ cell function associated with FSH elevation and oligozoospermia and impaired sperm motility and development of testicular adrenal rest tumours (30). Similarly, men with partial loss-of-function variants in *STAR* often progress through normal puberty, but can later show signs of gonadal dysfunction (14). Even though our patient has progressed through puberty normally, he needs to be monitored for later development of gonadal dysfunction and possible reduction in other adrenocortical hormones including mineralocorticoids, highlighting the importance of making a specific diagnosis in individuals with PAI (31).

### Limitations of the Study

*In vitro* functional studies to determine whether the combination of the three variants together reduce function to a level where steroidogenesis is not supported were not possible. To try and recapitulate even the effects of one variant in a transient or short-term cellular system only really gives a snapshot – hence trying to combine the effects of these variants in a biologically relevant system would be very challenging.

We cannot rule out the possibility that an, as yet, unidentified genetic cause of adrenal insufficiency is causative in this case but the finding of three variants in two known causal genes is persuasive that this could represent the first description of digenic, tri-allelic PAI.

## Author Disclaimer

The views expressed are those of the authors and not necessarily those of the National Health Service, National Institute for Health Research, or Department of Health.

## Data Availability Statement

The original contributions presented in the study are included in the article. Further inquiries can be directed to the corresponding authors.

## Author Contributions

NA clinically and biochemically evaluated the patient and wrote the first draft of the article. LM conceptualized the article. LM and AM did the molecular analysis. FB and JA contributed to the molecular evaluation and concept of the article. All authors contributed to manuscript revision, read and approved the submitted version.

## Funding

LM received the Medical Research Council UK Project Grant MR/K020455/1. JA is a Wellcome Trust Senior Research Fellow in Clinical Science (grants 098513/Z/12/Z and 209328/Z/17/Z) with research support from Great Ormond Street Hospital Children’s Charity (grant V2518) and the National Institute for Health Research, Great Ormond Street Hospital Biomedical Research Centre (grant IS-BRC-1215-20012).

## Conflict of Interest

The authors declare that the research was conducted in the absence of any commercial or financial relationships that could be construed as a potential conflict of interest.

## Publisher’s Note

All claims expressed in this article are solely those of the authors and do not necessarily represent those of their affiliated organizations, or those of the publisher, the editors and the reviewers. Any product that may be evaluated in this article, or claim that may be made by its manufacturer, is not guaranteed or endorsed by the publisher.
